# Perforated Small Bowel Adenocarcinoma: An Interesting Presentation of a Rare Disease

**DOI:** 10.7759/cureus.45094

**Published:** 2023-09-12

**Authors:** Toufeeq Suliman, Errington C Thompson, David A Denning

**Affiliations:** 1 General Surgery, Marshall University Joan C. Edwards School of Medicine, Huntington, USA; 2 Trauma and Surgical Critical Care, Marshall University Joan C. Edwards School of Medicine, Huntington, USA

**Keywords:** small bowel adenocarcinoma, anastomotic leak after gastrointestinal surgery, crohns, small bowel perforation, small bowel malignancy, inflammatory bowel disease

## Abstract

Primary small bowel adenocarcinoma (SBA) is a rare disease with no clear guidelines on screening, diagnosis, or treatment. It has been associated with hereditary cancer syndromes; however, most cases are sporadic and frequently associated with inflammatory bowel disease.

We present the case of a 43-year-old male who presented with abdominal pain, nausea, and vomiting and was initially diagnosed with a Crohn’s disease flare. He subsequently developed a small bowel perforation and was taken to the operating room for resection of the inflamed segment of the ileum; this was later found to be secondary to an obstructing small bowel adenocarcinoma. He developed an anastomotic leak, which mandated re-exploration and allowed for the completion of an oncologic resection.

Small bowel adenocarcinoma is difficult to diagnose and treat due to the absence of clear guidelines and the lack of randomized controlled trials in the setting of a very low incidence.

## Introduction

Primary small bowel malignancy is a rare disease with increasing incidence; it often presents with non-specific symptoms and is difficult to diagnose radiologically, making diagnosis challenging [1-2]. Of these malignancies, small bowel adenocarcinoma (SBA) and neuroendocrine tumors are the two most common etiologies, each accounting for 35%-40% of primary small bowel neoplasms [1], with the ileum being the least common location [3]. While inherited cancer syndromes increase the risk of developing SBA, most cases are sporadic and occur in patients with inflammatory bowel disease [4]. Small bowel adenocarcinomas usually present later in life, with a median age of 60 years at diagnosis [1], typically presenting with non-specific abdominal pain, nausea, vomiting, anemia-gastrointestinal bleeding, and/or weight loss [5].

Due to the rarity of this disease, there are no screening guidelines for SBA in Crohn’s disease; however, it is recommended to perform colonoscopy with biopsies every three years, starting eight years after diagnosis or at 45 years of age, whichever occurs earlier [6]. It is also recommended that the ileum be intubated during colonoscopy in the setting of inflammatory bowel disease [7].

There is also no definitive guideline regarding treatment; however, like colon cancer, non-metastatic SBA is typically treated by surgical resection with at least 5 cm of proximal and distal margins, as well as resection of adjacent mesentery and at least 10-15 nodes [1]. Small bowel adenocarcinoma involving the distal ileum should be treated with ileocecectomy or right hemicolectomy and associated mesentery [8].

As expected, the prognosis is significantly worse in late-stage disease, with five-year overall survival rates of 55%, 50%, 30%, and 5% for stage one, two, three, and four diseases, respectively [1, 9].

## Case presentation

**Figure 1:**
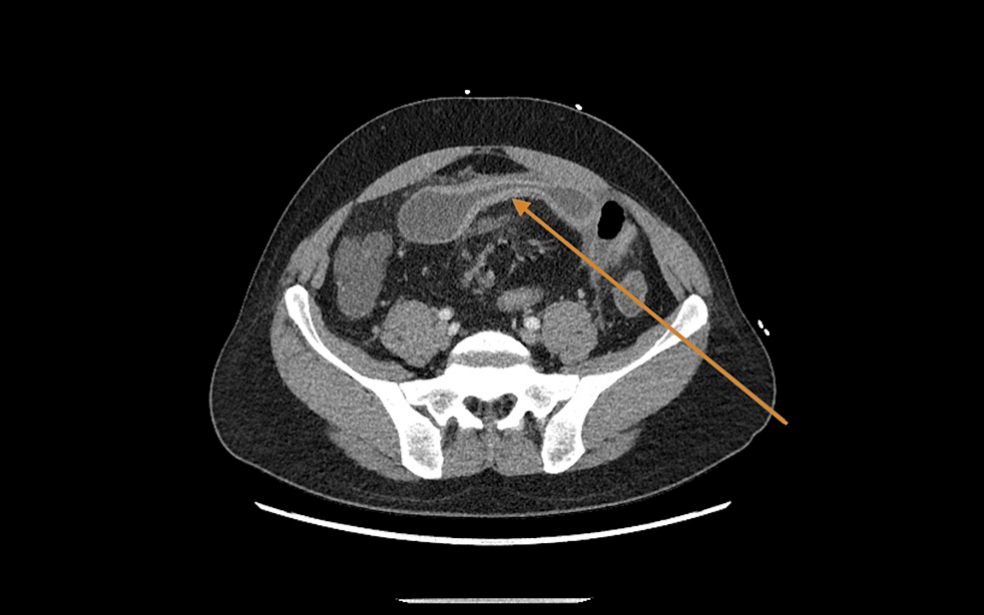
Computed tomography imaging shows an inflamed distal ileum (arrow) consistent with Crohn’s flare.

We present the case of a 43-year-old male with a known history of Crohn’s disease and no significant surgical history who initially presented to our emergency department with one day of abdominal pain as well as nausea and one episode of vomiting, with no change in bowel habits or blood in his stool. At this time, he was found to have leukocytosis (WBC: 21,400 per microliter) and a soft, distended, mildly tender abdomen. Computed tomography imaging showed wall thickening of the ileum and adjacent mesenteric edema/lymphadenopathy, suggesting a Crohn’s flare (Figure 1).

He was not receiving any treatment at the time and reported similar episodes approximately once per year since his initial diagnosis, treated with steroids. He was unable to follow up previously due to social reasons and has never been on maintenance or biological therapy. He had not had a colonoscopy since his initial diagnosis (based on a colonoscopy with biopsy) approximately 10 years prior to presentation.

**Figure 2:**
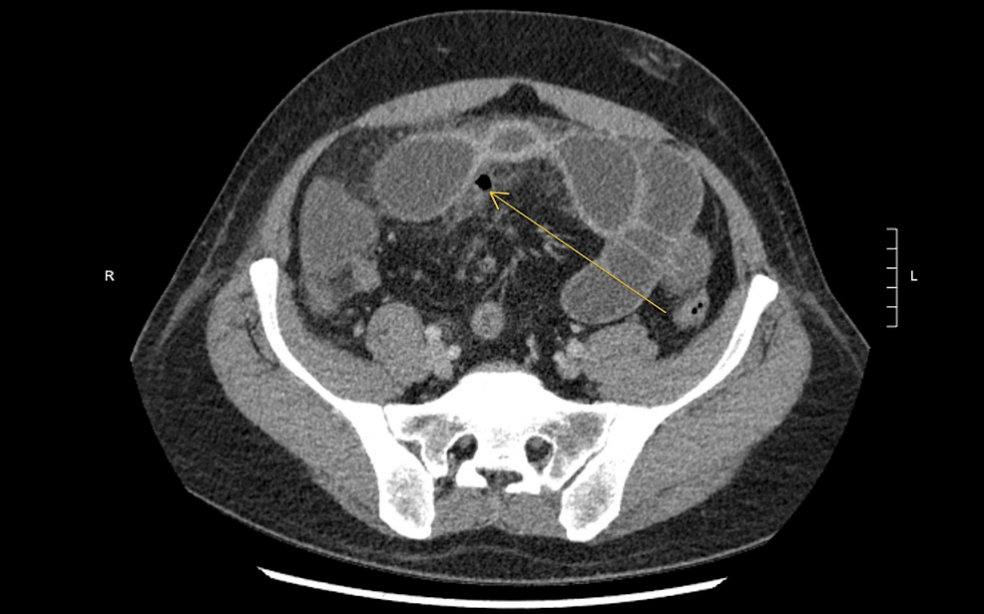
Computed tomography imaging shows worsening inflammation of the ileum with extraluminal air (arrow) and concern for obstruction.

He was admitted to the medical service with a gastroenterology consult and treated with intravenous steroids (methylprednisolone). The patient improved clinically with resolution of nausea and improvement in abdominal pain/exam and leukocytosis (18,500 per microliter) despite steroid therapy. He was scheduled for a colonoscopy by the gastroenterology service and was receiving polyethylene glycol as bowel preparation on hospital day three. During this time, he developed worsening abdominal pain, fever, and tachycardia. The abdomen was soft and distended, with generalized tenderness to palpation and voluntary guarding. A repeat CT scan was obtained, showing concern for a small bowel obstruction secondary to worsening inflammation of the ileum with extraluminal free air concerning a perforation (Figure 2).

At this time, it was suspected that the patient developed perforation of the small bowel secondary to the stress of an aggressive bowel regimen in the setting of active inflammatory bowel disease.

He was taken to the operating room for an exploratory laparotomy, where he was found to have an inflamed, thickened distal ileum with a friable mesentery and surrounding free fluid but no gross contamination. The distal ileum was resected, and an anti-peristaltic stapled side-to-side ileocolic anastomosis was performed. He was left intubated and transferred to the intensive care unit. The patient gradually improved and was extubated on post-operative day two with a return of bowel function. Unfortunately, on post-operative day three, he developed worsening abdominal pain and tachycardia, with a clinical exam consistent with peritonitis. He was taken back to the operative room for an exploratory laparotomy, where he was found to have an anastomotic leak at the site of the closure of the common enterotomy. Due to an anastomotic leak in the setting of contamination and reactive inflammation of the small bowel, the anastomosis was resected, and an end-ileostomy was matured. He was once again left intubated and taken to the intensive care unit.

Pathology from his initial resection showed a 4.1 cm invasive, grade two small bowel adenocarcinoma with invasion into the subserosa and over 15 negative lymph nodes (T3N0 disease) with a 29 cm proximal margin and 2.7 cm distal margin, along with granulomatous enteritis and areas of transmural inflammation suggesting Crohn's disease. These results were not available at the time of his second surgery. Pathology from the second resection showed no evidence of malignancy and an additional 5.7 cm of colon (distal margin). Based on this new information, it appeared that while he did have a diagnosis of Crohn’s disease, his presentation was likely secondary to a partially obstructing adenocarcinoma of the ileum.

He had a prolonged hospital course as expected but did slowly progress appropriately after his second surgery. He was extubated on post-operative day three (after the second surgery), his ileostomy began to function, and his diet slowly advanced. The patient required drainage of a transudative pleural effusion as well as interventional radiology consultation for CT-guided drainage of an intra-abdominal abscess on post-operative day 10 and was discharged home on post-operative day 17 after a course of antibiotics and physical/occupational therapy.

He has undergone CT of the chest, abdomen, and pelvis with no concern for metastatic disease and will undergo colonoscopy post-operatively, as well as be referred to medical oncology for possible adjuvant chemotherapy and gastroenterology for possible maintenance or biologic therapy for Crohn's disease.

## Discussion

Small bowel adenocarcinoma is a rare disease with limited evidence-based guidelines for screening, diagnosis, and treatment. We have a 43-year-old patient with a history of Crohn’s disease, which is known to be associated with SBA. Colon cancer screening guidelines would have recommended a colonoscopy approximately two years prior to his presentation, ideally with intubation of the ileum, which may have yielded an earlier diagnosis of his condition. Due to the similarities in presentation between SBA and a Crohn’s disease flare, with the latter being far more common, he was initially incorrectly diagnosed.

While the cause of the perforation could not be definitively established, the mechanical bowel preparation may have led to the perforation requiring his initial surgery. One could argue against colonoscopy in the setting of an acute Crohn’s disease flare; however, this patient had been lost to follow-up several times and was already significantly overdue on screening for colonoscopy, which may have impacted the decision to proceed with colonoscopy. Colonoscopy is a frequently performed screening, diagnostic, and therapeutic tool. There is an inherent risk of perforation, which occurs more frequently in patients with inflammatory bowel disease and those on steroid therapy, with a perforation rate of approximately 1% in patients with inflammatory bowel disease (as opposed to 0.6% in the general population) [10]. There is very limited data on perforation occurring during mechanical bowel prep; however, it is contraindicated in obstruction and severe colitis [11].

This patient was initially treated based on a working diagnosis of small bowel perforation secondary to inflammatory bowel disease, which led to inadequate margins at the time of the initial resection. This may have been diagnosed at the time of surgery had the specimen been dissected open on the back table in the operating room; however, there is no clear guideline or indication to do so. While his perforation was unfortunate and contributed to increased morbidity and length of stay, it did allow for further resection of the colon (distal margin) to an adequate oncologic resection (5 cm proximal and distal margin).

There was no gross perforation at the time of initial surgery; however, the extraluminal air seen on CT imaging could be used as an argument for upstaging this tumor and changing his treatment course with a lower threshold to pursue adjuvant chemotherapy. Based on his stage two disease, he would be a candidate for observation versus adjuvant therapy in the setting of curative surgery.

Adjuvant chemotherapy remains controversial, again due to the rarity of this disease; however, general consensus suggests observation for stage one or low-risk stage two disease after curative surgery and adjuvant chemotherapy for high-risk stage two and any stage three or four disease, with studies showing significant improvement in survival noted with the addition of adjuvant therapy in stage three disease [1]. The National Comprehensive Cancer Network (NCCN) suggests 5-fluorouracil with leucovorin (5FU/LV) or capecitabine for early-stage disease requiring adjuvant treatment and either FOLFOX (folinic acid, fluorouracil, oxaliplatin), CAPEOX (capecitabine, oxaliplatin), or 5FU/LV for three to six months for higher-stage disease [12]. The patient has been referred to medical oncology for evaluation and possible adjuvant therapy with the hope of improving overall survival.

## Conclusions

We present the case of a 43-year-old male with perforated SBA, likely secondary to mechanical bowel prep, in the setting of a partially obstructing tumor misdiagnosed as a Crohn’s flare. Due to the low incidence of this disease, there are few randomized controlled trials and therefore limited evidence-based guidelines on screening and treatment; as such, each case is considered independently. While he initially had an inadequate resection, he did require an operative takeback due to an anastomotic leak, which allowed for a complete oncologic resection. He has progressed well despite a prolonged hospital course and will require colonoscopy and adjuvant chemotherapy once healed from surgery.
